# Combining bacteriophages and essential oils for the elimination of monophasic *Salmonella* Typhimurium and evaluation of phage persistence in poultry farm environments

**DOI:** 10.1016/j.psj.2026.107124

**Published:** 2026-05-13

**Authors:** N. Gentile, L. Montoro-Dasi, A. Marco-Fuertes, P.J. Marín-García, P. Domingo-Calap, L. Lorenzo-Rebenaque, C. Marin

**Affiliations:** a Facultad de Veterinaria, Instituto de Ciencias Biomédicas, Universidad Cardenal Herrera-CEU, CEU Universities, Alfara del Patriarca, 46115 Valencia, Spain; b Institute of Science and Animal Technology, Universitat Politècnica de València, Camí de Vera s/n, 46022 València, España, Spain; c Department of Animal Production and Health, Veterinary Public Health and Food Science and Technology (PASAPTA), Facultad de Veterinaria, Universidad Cardenal Herrera-CEU, CEU Universities, Valencia, Spain; d Department of Genetics, Universitat de València, 46100 Burjassot, Valencia, Spain; e Institute for Integrative Systems Biology (I2SysBio), Universitat de València-CSIC, 46980 Paterna, Valencia, Spain

**Keywords:** Bacteriophages, Essential oils, Cleaning and disinfection protocols, Poultry farms

## Abstract

*Salmonella enterica* serovar Typhimurium, monophasic variant (mST) and non-target species, such as *E. coli*, remain a major challenge for poultry production systems, particularly due to their ability to form biofilms and persist in farm environments and their increasing resistance to conventional chemical disinfectants. The present study aimed to develop and evaluate an innovative, sustainable, and eco-friendly cleaning and disinfection protocol using a combination of an essential oil (EO) and a bacteriophage (BP) cocktail under conditions simulating a poultry farming environment to eliminate persistent bacteria. Preliminary *in vitro* assays were conducted to identify the most effective EO against a target strain, selecting carvacrol as the disinfectant. In addition, three bacteriophages were selected based on their lytic spectrum against mST. The cleaning and disinfection (C&D) protocol was applied in an experimental poultry house following natural environmental contamination generated after experimental infection of the animals with mST. The protocol consisted of five sequential steps, ranging from mechanical removal of organic matter and detergent application to the disinfection phases (EO + BP). The results showed a progressive reduction of *Salmonella* loads on all surfaces, with complete elimination after the second bacteriophage application (p < 0.001). A marked reduction in non-target species counts was also observed, indicating an overall improvement in environmental hygiene. Analysis of bacteriophage environmental persistence revealed detectable levels up to one week after application, suggesting short-term environmental stability. Furthermore, the combined use of carvacrol and bacteriophages proved to be an effective and sustainable alternative to conventional chemical disinfectants, representing a promising green strategy for *Salmonella* control in poultry farm environments.

## Introduction

*Salmonella enterica* remains one of the major zoonotic pathogens, posing a persistent challenge to public health and food safety worldwide (EFSA, 2025). According to the European Food Safety Authority's (**EFSA**) 2024 report, 79,703 confirmed cases of human salmonellosis were reported in the European Union (**EU**), corresponding to a notification rate of 18.0 cases per 100,000 population (EFSA, 2025). This represented a 16.9 % increase compared to 2022, with an estimated annual economic burden of approximately 3 billion euros. Among the top five EU-acquired *Salmonella* serovars involved in human infections, *Salmonella enterica* serovar Typhimurium and its monophasic variant (**mST** 1,4,[5],12:i:) are of particular concern due to their high prevalence in intensive swine and poultry production systems, as well as their frequent involvement in human outbreaks associated with contaminated food products (Parsons et al., 2013; EFSA, 2025). In 2024, mST ranked third among the most frequently isolated serovars, accounting for 10.4 % of the reported human cases, following *S.* Enteritidis (58.4 %) and *S.* Typhimurium (11 %) (EFSA, 2025). These trends reflect the progressive increase in antimicrobial resistance in *Salmonella* over the past decades (Wang et al., 2025).

In Europe, Regulation (EC) No. 2160/2003 mandates the implementation of National Control Programs (**NCPs**) for *Salmonella* serotypes considered of “public health significance”, to be applied at all stages of the poultry production chain, from rearing and processing to distribution (European Commission, 2003; Samper-Cativiela et al., 2023). Effective control of *Salmonella* at the farm level is therefore essential to interrupt transmission along the food production chain. Among available interventions, cleaning and disinfection (**C&D**) protocols play a key role in the early stages of production, contributing to reducing infection-related productivity losses and improving animal health (Papoula-Pereira et al., 2025).

Conventional C&D protocols are a cornerstone of farm biosecurity, but their effectiveness can be limited by the persistence of *Salmonella* in the farm environment, mainly due to its ability to form biofilms that protect bacteria from chemical disinfectants (Chylkova et al., 2017; Bezek et al., 2023; Gentile et al., 2025). A wide range of chemical disinfectants with different mechanisms of action are available and can be used alone or in combination to improve antimicrobial efficacy. However, their effectiveness depends on environmental factors such as surface type, temperature, organic matter and pH, and no single agent is effective under all conditions (Hansson et al., 2025; Gentile et al., 2025). Moreover, the excessive and repeated use of chemical disinfectants can have undesirable effects not only on the environment, promoting the development of microbial tolerance, but also on human health (Tong et al., 2021; Maillard and Pascoe, 2024; Obe et al., 2024). In recent years, C&D procedures have also been acknowledged as a health risk, due to prolonged exposure to disinfectant residues (Kumbhar and Geddugol, 2025). In the EU, the use of formaldehyde has been banned under Regulation (EC) No. 1223/2009, despite its proven efficacy against *Salmonella*, as it is classified as a carcinogenic substance for humans (European Commission, 2009).

For this reason, there is increasing interest in sustainable and eco-friendly based control measures that can complement or replace conventional C&D products (Gentile et al., 2025). Among these alternatives, the essential oils (**EOs**) are attracting renewed interest (Wang et al., 2023a). These are mixtures of aromatic compounds extracted from different parts of plants, of which approximately 300 are commercially available for several applications (Gentile et al., 2025). Due to their hydrophobic nature, they interact with bacterial membranes, disrupting their integrity and causing the loss of vital components, ultimately leading to cell death (Chouhan et al., 2017). Numerous studies have evaluated the antimicrobial activity of different EO against various *Salmonella* serovars (Gentile et al., 2025). However, in recent years, increasing attention has been directed toward assessing their applicability as biocides, particularly in the food industry.

Similarly, bacteriophages (**BP**) have emerged as natural and highly specific biocontrol agents against bacterial pathogens. These viruses lack the cellular structure required for independent survival and depend entirely on the bacterial host cell for replication (Jordá et al., 2023). Bacteriophages can selectively lyse *Salmonella* strains without affecting the environmental bacterial communities or in the microbiota of the animals, making them an interesting alternative to conventional chemical disinfectants (Sevilla-Navarro et al., 2024). They can be applied as single preparations or combined in cocktails to broaden their host range and enhance antibacterial efficacy. In recent years, different studies have demonstrated the effectiveness of BP cocktails against *Salmonella* under laboratory and field conditions, with variable results (Marin et al., 2018; Jordá et al., 2023; Sevilla-Navarro et al., 2024).

Despite the promising results obtained separately with both approaches, studies assessing the potential combination effect between EO and BP are still lacking, particularly those aimed not only at eliminating *Salmonella* but also at controlling other related *Enterobacteriaceae*, such as *E. coli,* under real poultry farming conditions. For this reason, the aim of this study was to develop and evaluate an innovative, specific, eco-friendly C&D protocol that integrates an EO and a BP cocktail to control mST in an experimental poultry farm under farm-like conditions. In addition, the presence of *E. coli* was evaluated as a microbiological indicator of hygiene across the entire C&D process. Finally, the study aimed to assess the environmental persistence and stability of the applied phages under farm conditions. To this end, preliminary *in vitro* assays were performed to identify the most effective EO and BP prior to their *in vivo* application.

## Materials and methods

### Infection strain

The strain mST (CEU-215S) used for infection of day-old chicks was previously isolated from avian fecal samples and stored at -80 °C at CEU-Cardenal Herrera University (**CEU-UCH**) until use (Marin et al., 2018).

Firstly, an antimicrobial susceptibility test was performed to characterize the antimicrobial resistance profile of the mST strain used for both experimental trials: Experiment 1 and Experiment 2. For this purpose, the EU Surveillance *Salmonella/E. coli* EUVSEC3 Sensititre Plate (Thermo Scientific Sensititre, Madrid, Spain) was used, which includes the following antimicrobials: amikacin (**AMI**), gentamicin (**GEN**), chloramphenicol (**CHL**), meropenem (**MER**), cefotaxime (**CTA**), ceftazidime (**CTZ**), sulfamethoxazole (**SME**), trimethoprim (**TRI**), tigecycline (**TIG**), azithromycin (**AZI**), ampicillin (**AMP**), colistin (**COL**), ciprofloxacin (**CIP**), nalidixic acid (**NAL**), and tetracycline (**TET**) (Marco-Fuertes et al., 2023). Sensititre plate results were analyzed according to the breakpoints established by the European Committee on Antimicrobial Susceptibility Testing (**EUCAST**) in 2025, and multidrug resistance (**MDR**) was defined as acquired resistance to at least one agent in three or more antimicrobial classes (EUCAST, 2024). The details of the antimicrobial panel and corresponding EUCAST breakpoint concentrations are reported in Supplementary Table S1. For this purpose, the mST strain was thawed and cultured on nutrient agar at 37 ± 1 °C for 24 h. Colonies were then suspended in 5 mL of sterile deionized water (T3339; ThermoFisher Scientific, Madrid, Spain) and adjusted to a 0.5 McFarland standard using a Nephelometer (ThermoFisher Scientific™, Madrid, Spain). Subsequently, 10 µL of the suspension were added to 11 mL of Mueller–Hinton broth (T3462; ThermoFisher Scientific™, Madrid, Spain), and 50 µL of this mixture were dispensed into each well of the Sensititre plate. The plate was sealed with parafilm and incubated at 37 ± 1 °C for 24 h, after which results were read manually using the Sensititre™ Vizion™ Digital MIC Viewing System (ThermoFisher Scientific, Madrid, Spain).

For subculture preparation, the mST strain was first streaked on selective chromogenic agar (**ASAP**; bioMérieux®, Marcy l’Étoile, France) and then incubated at 37 ± 1 °C for 24 ± 3 h to obtain isolated colonies. Subsequently, an individual colony was transferred to nutrient agar to promote rapid growth. From this culture, an overnight inoculum was prepared using buffered peptone water (**BPW** Scharlab®, Barcelona, Spain), and the bacterial concentration was standardized to an optical density (OD) of 0.2 at 600 nm (∼10^8^ CFU/mL) for the experimental trials.

### Experimental 1: in vitro trial

#### Essential oil selection

To assess the efficacy of EO against the infecting mST strain, eight commercial essential oils were selected and tested based on their documented antimicrobial activity (carvacrol, eugenol, rosemary oil, naringin, berberine hemisulfate salt, bergamot oil, cinnamaldehyde, and tea tree oil) (Wińska et al., 2019). All of them presented a purity of 99.9 % (Sigma-Aldrich Inc.), and the list of essential oils analyzed is given in Table 1. The organic solvent dimethyl sulfoxide (**DMSO**) used to dissolve the EO also had a purity of 99.9 % (Sigma-Aldrich Inc., ref. D5879).

**Table 1:** Commercial EOs and their concentrations used in the in vitro assays. The table reports the botanical name, essential oil, and corresponding concentrations (µg/mL) at three dilution levels (1 %, 0.5 %, and 0.25 %).

Botanical name	Essential oil	Density g/mL	µg/mL in 1 % dilution	µg/mL in 0.50 % dilution	µg/mL in 0.25 % dilution	T
*Aetheroleum Origani*	Carvacrol	0.976	9760	4880	2440	Sigma-W224511
*Syzygium aromaticum*	Eugenol	1.06	10600	5300	2650	Sigma-E51791
*Rosmarinus officinalis*	Rosemary oil	0.91	9100	4550	2275	Sigma-W299200
*Citrus fruit*	Naringin	0	0	0	0	Sigma-N1376
*Berberis vulgaris*	Berberine hemisulfate salt	0	0	0	0	Sigma-B3412
*Citrus bergamia Risso*	Bergamot oil	0.877	8770	4385	2192.5	Sigma - B4383
*Aetheroleum Cinnamomi*	Cinnamaldehyde	1.05	10500	5250	2625	Sigma-W228613
*Melaleuca alternifolia*	Tea tree oil	0.898	8980	4490	2245	Sigma-W390208

The effectiveness of the EO used for C&D was assessed through an *in vitro* susceptibility test. To this end, the minimum inhibitory concentrations (**MICs**) of the EO and DMSO were determined against the infecting strain. For each EO, three concentrations (1 %, 0.5 %, and 0.25 %) were tested, and both negative and positive controls were included. Moreover, the diluent DMSO was also evaluated for its potential toxic effects on bacteria. For this purpose, DMSO was preliminarily evaluated at three different concentrations (10 %, 1 %, and 0.1 %).

Antimicrobial activity testing of the EO and DMSO was conducted using plastic (virgin polystyrene) microtiter plates containing the extenders. In each well, the EO solution at one of the tested concentrations was added and was then inoculated with 100 µL of bacterial suspension at a final concentration of 10⁵ CFU/mL, prepared by mixing 10 µL of mST (10⁸ CFU/mL) with 90 µL of 1 % DMSO. Likewise, DMSO was also tested by inoculating 100 µL of bacterial suspension and 90 µL of DMSO at different concentrations (10 %, 1 %, and 0.1 %). The MIC was defined as the lowest concentration of EO at which visible bacterial growth was completely inhibited. To confirm the MIC, turbidity was evaluated, and the suspected well content was seeded onto ASAP. The test was performed in triplicate.

#### Bacteriophage selection

The BPs used in the C&D protocol are BPs belonging to the CEU-UCH phage library, and their effectiveness has been demonstrated against a broad spectrum of mST strains. The BPs selected were: Ф CEU1P2_0402 (propagation strain CEU-5S), Ф CEU9P2_2802 (propagation strain CEU-92S) and Ф CEU10P2_2802 (propagation strain CEU-97S).

For phage isolation, 50 mL of urban sewage water was collected and homogenized. Samples were centrifuged at 16,000 × g for 5 min, and the supernatant was then filtered through a 0.22 μm membrane. The filtered supernatant was used to detect the presence of phages by spot assay on different mST lawns, as described by Moreno Switt et al. (2015). Briefly, 200 μL of a log-phase bacterial culture in Luria-Bertani (**LB**; Shcarlab, Madrid, Spain), adjusted to an optical density at 600 nm (OD₆₀₀) of 0.2 (∼10⁸ CFU/mL), was mixed with 5 mL of LB soft agar (0.6 % agar) tempered to 50 °C and poured onto previously prepared and dried LB basal agar (1.6 % agar). Plates were allowed to dry in a laminar flow hood for 5 min. Subsequently, 20 μL of the sample was spotted onto the surface of the double-layer agar and incubated at 37 ± 1 °C for 24 h. The appearance of a clear zone on the bacterial lawn, resulting from host cell lysis, indicated the presence of a specific phage. Then, lysates of single plaques were mixed in Phosphate Buffered Saline diluent (**PBS**; Shcarlab, Madrid, Spain) and centrifuged at 5,000 x g for 5 min, and the supernatant was filtered through a 0.22 μm membrane. Each BP was purified by serial dilutions and plated on LB agar (**LB**; Shcarlab, Madrid, Spain). The process was repeated three times, after which the purified BP was stored at 4 °C.

Phages were selected for this study based on their lytic activity against the target bacterial strain (mST, CEU-215S), determined by spot assay as described above.

For the BP characterization, thermal and pH stability assays, a one-step growth assay, and genomic analysis were performed. Thermal stability was evaluated by incubating the BP at different temperatures (4, 37, 60, 80, and 100 °C), while pH stability was assessed across a pH range from 2 to 12, using HCl and NaOH (Fisher Scientific, Hampshire, UK). In both assays, BPs were subjected to different temperature and pH conditions and incubated for 1 h. After incubation, BPs concentrations were determined by serially diluting the samples (1:10) and plating them using the drop assay method in LB agar, followed by incubation at 37 ± 1 °C for 24 h. The rates of BPs pH/thermal stability were calculated using the formula: BP stability rate (%) = BP concentration (PFU/mL) under certain conditions/initial BP concentration added (PFU/mL) × 100 %. These experiments were performed three times. The one-step growth assay was carried out using an overnight culture of *Salmonella* cultures (10⁸ CFU/mL), and BP was added at a multiplicity of infection (**MOI**) of 0.1. The mixture was incubated at 37 °C for 5 mins to allow phage adsorption. Following incubation, the sample was centrifuged at 12,000 g at 4 °C for 2 mins, and the supernatant was removed to eliminate free BPs and prevent a new infection cycle. The pellet, containing infected bacteria, was resuspended in 10 mL of LB broth and incubated at 37 °C for 1 h. Aliquots were taken at 5, 10, 15, 20, 30, 40, 50, and 60 mins. Each aliquot was centrifuged at 12,000 g for 2 mins, and the supernatant was collected and serially diluted (1:10) for plating using the drop assay method. The experiment was performed in triplicate.

For genomic characterization, phages were sequenced in the Foundation for the Promotion of Health and Biomedical Research of the Valencian Community (**FISABIO**). To this end, DNA isolation and genome sequencing were carried out according to Domingo-Calap et al. (2020). To this end, the phages were treated with DNAse I to remove non-encapsidated DNA, followed by extraction using a commercial kit (QIAamp Viral RNA Mini Kit, QIAGEN, Hilden, Germany). Finally, DNA was sequenced with an Illumina MiSeq. Reads were screened for contaminants prior to de novo assembly using Kraken 276. Then, Taxonomic Profiling and Read Pre-processing were performed*.* Raw reads were quality checked using fastp program (Chen et al., 2018), applying default filtering and tail trimming (–cut_window_size 10, –cut_mean_quality 20). Reads passing the quality filters were subjected to taxonomic classification, estimating contaminations using KMA (Clausen et al., 2018) v1.4.15 against the NCBI NT database, with phylogenetic aggregation performed by CCMetagen (Marcelino et al., 2020) v1.4.1. To focus on the viral fraction, host-derived reads, if any, were depleted by mapping against the *Salmonella enterica* reference genome (subsp. *enterica* serovar Typhimurium isolate VNB151-sc-2315230, Accession LT795114.1) using BWA-MEM (Li and Durbin, 2009) v0.7.17- -r1188. Unmapped (non-host) reads were extracted and converted to FASTQ format using SAMtools (Li et al., 2009) v1.20 and bedtools (Quinlan, 2014) v2.30.0 for downstream de novo assembly. Filtered viral reads were downsampled using seqtk (GitHub, 2025) v1.4-r122 program. Finally, viral reads were assembled using SPAdes (Bankevich et al., 2012) v4.2.0 with the –metaviral algorithm, employing a multi-k-mer strategy (21, 33, 55, 77, 99, 127). Assembled contigs were filtered to retain only sequences >5,000 bp. Genomes were then submitted to a first round of annotation using Pharokka (Bouras et al., 2023) v1.8.2 for functional assignment. The Terminase Large Subunit (**TerL**) gene was then used to orient the phage’s genomes during a second round of pharokka annotation. Genomic quality, including completeness, contamination, and provirus status, was assessed using CheckV v1.0.3. (Nayfach et al., 2021). Completeness was estimated based on the Average Amino Acid Identity (**AAI**) against the CheckV reference database. Standardized GenBank files were utilized for comparative genomic analysis. Whole-genome synteny and protein-level homology were evaluated using clinker v0.0.32. (Gilchrist and Chooi, 2021). Alignment blocks were calculated using a 30% identity threshold to visualize conserved functional modules across the isolates and against the reference *Salmonella* phage INT59.

### Experimental 2: cleaning and disinfection procedure with a combination of essential oil and bacteriophages

The procedure was conducted in accordance with the protocol approved by the Directorate-General for Agriculture, Fisheries, and Livestock of the Valencian Community (research code: 2025-VSC-PEA-0097) and was carried out in the animal facilities of the Universitat Politècnica de València (**UPV**, Valencia, Spain; ES462500001091).

#### Animal housing

A total of 60 one-day-old Specific Pathogen Free (**SPF**) chicks were housed in an experimental house in two identical pens (30 animals/pen) and reared for 28 days to promote natural environmental contamination through *Salmonella* shedding, according to specific guidelines of the European Pharmacopoeia (EP): 04/2013:2520 and 04/2013:2521. This setup simulated pathogen persistence in poultry house environments prior to the application of C&D procedures. Animals were managed according to standard poultry production practices. Each pen measured 1.725 m² of floor area and 4.575 m² of wall surface and was equipped with wood shavings as litter, programmable lighting, automatic heating control, and humidity regulation. The ambient temperature was gradually reduced from 34 °C at arrival to 24 °C by day 28 of life. Animals had free access to water and feed throughout the experimental period. To assess the animals' health status, their weight was measured weekly, and water consumption was monitored throughout the experiment.

#### Salmonella infection and excretion

**Fig. 1:**
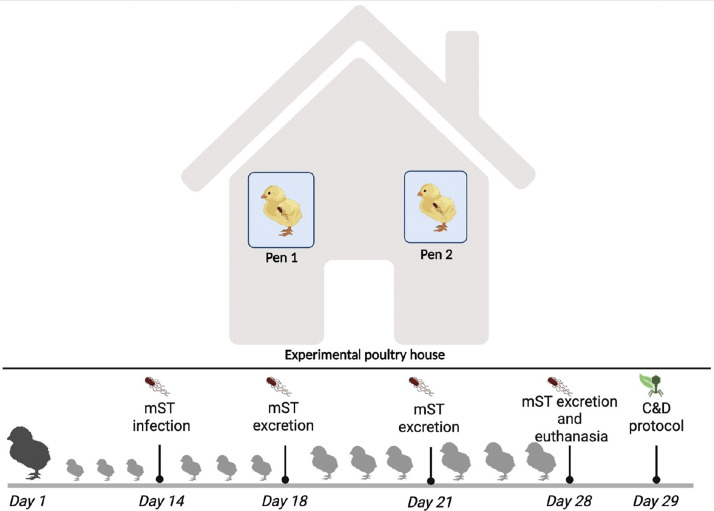
Schematic representation of the experimental poultry house and trial timeline. The poultry house was divided into two pens (Pen 1 and Pen 2), each housing a group of chickens. The experimental timeline included chick placement on day 1, oral infection with mST on day 14, the monitoring of *Salmonella* excretion on days 18, 21 and 28, animal euthanasia on day 28, and implementation of the C&D protocol on day 29 (Created in BioRender. GENTILE, N. (2026) https://BioRender.com/vrlwiiw).

With the aim of achieving natural environmental *Salmonella* contamination through the excretion of *Salmonella* in animal feces, all SPF chicks were orally challenged with CEU-215S mST strain at 14 days of age (10⁵ CFU/mL per chick). Starting on day 4 post-infection (18 days of age), *Salmonella* shedding in the environment was monitored weekly at 21 and 28 days of age (corresponding to 7 and 14 days post-infection, respectively), according to specific guidelines of the EP: 04/2013:2520 and 04/2013:2521. Finally, at 28 days of age, the animals were euthanized and the pens cleaned and disinfected (day 29) according to the C&D ingredients selected in Experiment 1 (Fig. 1).

#### Cleaning and disinfection procedures

Firstly, the most effective EO selected in Experiment 1 (carvacrol) was prepared for use. Carvacrol was initially diluted 1:1 (vol/vol) in DMSO and subsequently diluted with water to obtain a final concentration of 1 %, according to Campana and Baffone (2018). Secondly, the BP cocktail was prepared by mixing the selected BPs at equal titers and volumes and then diluting the cocktail with water to achieve a final concentration of 10⁷ PFU/mL, according to previous studies (Korzeniowski et al., 2022; Gvaladze et al., 2024). All solutions were freshly prepared on the day of use.

The day after the animals were euthanized, the C&D protocol (EO+BP) was applied in the experimental poultry facilities. The C&D protocol included five phases. First (i) removal of organic matter using high-pressure water, followed (ii) by the application of foaming detergent (Hyprelva Net Plus, 3 % following manufacturer’s recommendations) sprayed over all surfaces. After 20 mins of contact time, the surfaces were washed and left to dry following the manufacturer's recommendation. Then, (iii) disinfection was performed using a 1 % solution of carvacrol. The disinfectant solution was aerosolized using a fogger (Mgła-E, Poltech Corp., Warsaw, Poland), generating particles smaller than 50 μm, and allowed to act for one hour. Subsequently, (iv) the BP cocktail (10⁷ PFU/mL) was aerosolized using a fogging device, (v) followed by a second identical BP application 24 h later (Table 2).

**Table 2:** Steps of C&D protocol.

Phase	Treatment	Product / Method	Purpose	Conditions
**I**	Removal of organic matter	High-pressure water washing	Elimination of visible organic material prior to sanitation	Applied to all surfaces;
**II**	Detergent cleaning	Foaming detergent (Hyprelva Net Plus, 3%)	Removal of residual organic matter	Applied to all surfaces;
**20 min contact time**				
Surfaces subsequently rinsed and allowed to dry				
**III**	Disinfection with EO	Carvacrol solution (1%)	Reduction of bacterial load	Aerosolized to all surfaces using a fogger (Mgła-E, Poltech Corp., Warsaw, Poland) with particle size <50 μm;
**1 h contact time**				
**IV**	Disinfection with BP_C_	First dose of BP_C_ (10⁷ PFU/mL)	Targeted reduction or elimination of mST contamination	Aerosolized to all surfaces using a fogger (Mgła-E, Poltech Corp., Warsaw, Poland) with particle size <50 μm;
**24 h contact time**				
**V**	Disinfection with BP_C_	Second dose of BP_C_ (10⁷ PFU/mL)	Reinforcement of bacteriophage activity against mST	Aerosolized to all surfaces using a fogging device (Mgła-E, Poltech Corp., Warsaw, Poland) with particle size <50 μm;

**BP_C_**: Bacteriophage Cocktail

#### Sampling

To assess the *Salmonella* status of the experimental poultry houses before animal arrival, environmental samples were collected. Thus, samples were collected from the floor (n=2), the walls (n=2), the in-house mobile (n=4), and the hallways (n=2). Samples were collected using 12 mL Stericloth® neutralizing wipes with 10% Peptone Water (Bioser, Barcelona, Spain). On animal arrival, the *Salmonella* status of the day-old chick flock was assessed by collecting meconium and delivery box liners from the shipping boxes. Moreover, after chicken infection (14 days of rearing), cloacal swabs were collected from all animals to assess *Salmonella* excretion at 18, 21, and 28 days of age (corresponding to 4, 7, and 14 days post-infection).

To assess the C&D procedure, surface samples were collected from walls (n=2), floors (n=2), and floor-wall junctions (n=2) at five different time points: before starting C&D protocol (**T0**), after the foaming detergent (**T1**), after carvacrol application (**T2**), 24 h after the first BP cocktail application (**T3**), and 4 h after the second BP cocktail application (**T4**). Sampling was performed by wiping 1 m² surface area using both sides of a sterile wipe (Whirl-Pak®, Scharlab, Madrid). Finally, to monitor BP stability in the environment, samples from walls, floors, and floor-wall junctions were collected one day and one week after conclusion of the C&D procedure, as reported above.

### Laboratory analysis

#### Salmonella excretion assessment

To assess *Salmonella* excretion in feces, cloacal swabs collected were analyzed according to ISO/TS 6579-1:2017. Briefly, samples were pre-enriched in BPW at a 1:10 (vol/vol) and incubated at 37 ± 1 °C for 18 ± 2 h. Pre-enriched samples were then inoculated onto Modified Semi-Solid Rappaport Vassiliadis agar plates (**MSRV**, bioMérieux®, Marcy l’Étoile, France) and incubated at 41.5 ± 1 °C for 48 h. Plates showing suspected growth were then transferred to ASAP, and incubated at 37 ± 1 °C for 24 ± 3 h. To confirm the results, a biochemical test (API-20E, bioMérieux®, Marcy l’Étoile, France) was performed.

#### Environmental Enterobacteriaceae assessment

To assess C&D procedure effectiveness, environmental wipes collected were individually transferred into sterile bags, diluted 1:10 in BPW, and homogenized for 5 min using a Laboratory Blender (Stomacher 400, Seward Limited, West Sussex, UK). Once diluted, *Salmonella* and *E. coli* count was performed. For this purpose, a serial dilution of the samples was prepared, and 100 µL of each dilution was plated onto *Salmonella* agar (ASAP) and chromogenic selective *E. coli* agar (Tryptone Bile X-Glucuronic Agar, **TBX**, Scharlab®, Madrid). Then, plates were incubated at 37 ± 1 °C for 24 ± 3 h. After incubation, colonies were counted, and the corresponding CFU/mL values were determined.

#### Environmental bacteriophages stability

To assess the environmental BP stability, the BP count was performed. Briefly, 1 mL of the environmental wipes collected as reported above was diluted 1:10 in LB broth, thoroughly homogenized, centrifuged (8,000 x g at 4 °C for 10 min), and filtered through a 0.45 µm and 0.22 µm filter. Serial dilutions were then prepared in LB. Then, 200 μL of a log-phase culture of the bacterial suspensions in LB at an optical density (OD) 600 nm of 0.2 (∼10^8^ CFU/mL) was added to 5 mL of LB agar (LB with 0.6 % agar) tempered to 50 °C and poured onto previously prepared and dried LB basal agar (with 1.6 % agar). The plates were dried in a laminar flow hood for 5 min. Subsequently, 10 μL of the sample suspension was spotted onto the surface of the double-layer agar and incubated at 37 ± 1 °C for 24 ± 3 h. BP titration was performed in triplicate.

### Statistical analysis

For the *in vitro* trial, a descriptive analysis was conducted to determine the MIC of the infection strain against EO and DMSO. Regarding the *in vivo* trial, a Generalized Linear Model (**GLM**) with a binomial distribution was applied to analyze *Salmonella* excretion throughout the rearing period. To assess the effectiveness of the C&D protocol, *Salmonella* and *E. coli* count (log₁₀ CFU/m²) at different time points and the persistence of BP count (log₁₀ PFU/g) in the environment, a GLM was used after logarithmic (log_10_) transformation of the data. Statistical significance was set at *p* < 0.05. All analyses were performed using SPSS software version 27.0 (SPSS Inc., Chicago, IL, USA).

## Results

### Antimicrobial susceptibility test of monophasic *Salmonella* Typhimurium strain

The mST strain used in both experimental trials was resistant to 3 of the 11 antimicrobial families analyzed and was classified as MDR. Its AMR profile showed resistance to AMP, SME and TET, while it remained susceptible to the remaining antimicrobials tested.

### Experimental 1: in vitro trial

#### Antimicrobial susceptibility test of essential oils

The antibacterial activity of the tested products was evaluated. Among the EO tested, carvacrol, eugenol, and berberine hemisulfate salt showed the highest efficacy against mST, achieving complete inhibition (100 %) at the lowest concentration (0.25 %). Cinnamaldehyde and rosemary exhibited significant inhibitory activity at 1 %, while tea tree oil was effective at 0.5 %. In contrast, naringin and bergamot oil showed no efficacy against mST. The MIC of each EOs has been reported in Table 3. Moreover, DMSO exhibited no antitoxic activity at the tested concentrations.

**Table 3:** Minimum inhibitory concentration (MIC) of eight EOs against mST expressed in percentage (%).

MIC								
*Salmonella* serotype	Carvacrol (%)	Eugenol (%)	Rosmery oil (%)	Naringin (%)	Berberine (%)	Bergamot oil (%)	Cinnamaldehyde (%)	Tea tree oil (%)
mST	0.25	0.25	1	>1	0.25	>1	1	0.5

#### Bacteriophage lytic activity

The lytic spectrum of the three BPs included in the cocktail, Ф CEU1αP2_0402, Ф CEU9αP2_2802, and Ф CEU10αP2_2802, was evaluated against the *Salmonella* strain CEU-215S. BP Ф CEU1αP2_0402 and Ф CEU10αP2_2802 exhibited strong lytic activity against the target strain, producing well-defined and transparent plaques on the bacterial lawn, indicative of a lytic nature. In contrast, Ф CEU9αP2_2802 showed comparatively weaker lytic activity.

**Fig. 2:**
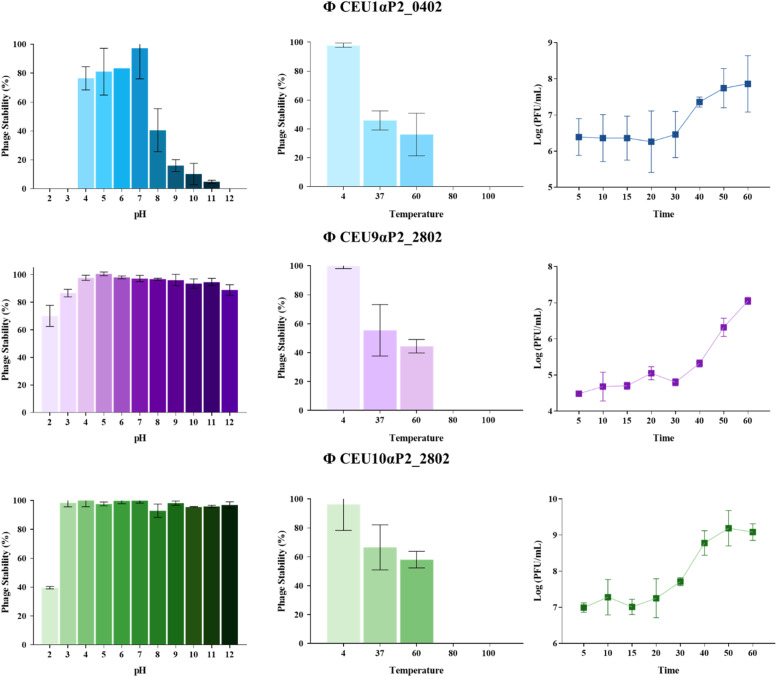
Stability of the BP under different stress conditions (pH stability and thermal stability) and one-step growth curves for ΦCEU1P2_0402, ΦCEU9P2_2802, and ΦCEU10P2_2802. Data are presented as means ± standard error from three independent experiments. Error bars indicate the standard error of the mean.

The results of BP characterization showed that all three phages remained stable after 1 h of exposure to pH values ranging from 4 to 11 and at 4 °C, while phage titers decreased between 37 °C and 60 °C and complete inactivation was observed at temperatures above 80 °C. One-step growth assays revealed eclipse periods of approximately 30 min for ΦCEU1P2_0402 and ΦCEU9P2_2802, and 20 min for ΦCEU10P2_2802. Fig. 2 shows the stability of the BP under different stress conditions and one-step growth curves.

Genomic analysis confirmed three high-quality phage genomes with complete circular structures: ΦCEU1P2_0402 (50,542 bp) and ΦCEU9P2_2802 and ΦCEU10P2_2802 (45,056 bp). In addition, CheckV results show that all three isolates were classified as high-quality viral genomes, with no detectable host contamination, representing two distinct genomic clusters of *Salmonella*-associated BP. Finally, strains ΦCEU9P2_2802 and ΦCEU10P2_2802 were found to be homologs of *Salmonella* INT59 phage, the ΦCEU1P2_0402 was not clearly classified, whereas blast searches were showing partial matches with *Pseudomonas* spp. prophages.

### Experimental 2: in vivo trial

At the beginning of the study, the negative *Salmonella* status of the experimental houses and the day-old chick flock was confirmed.

#### Salmonella detection

Following infection, the animals shed the bacterium continuously throughout the rearing period (100 % of animals shed the bacterium at 4, 7, and 14 days post-infection). Moreover, no clinical signs, episodes of diarrhea, or mortality were observed in the animals during the rearing period.

#### Cleaning and disinfection application

Regarding *Salmonella* count, the data show a progressive reduction in bacterial loads across all surfaces. In particular, on walls and wall-floor junctions, a rapid decrease in bacterial counts was observed at T2 (after carvacrol application), with mean values of 0 and 2.8 log₁₀ CFU/m², respectively (p-value < 0.001). In contrast, the floor showed a greater persistence of contamination, with detectable bacterial counts remaining until T3 (after the first application of the BP cocktail). At T4, *Salmonella* was eliminated from all surfaces (p < 0.001) (Table 4).

**Table 4:** *Salmonella* count at different time points (log_10_ CFU/m^2^), expressed as mean ± standard error (SE).

Surface	Concentration of *Salmonella* (log_10_ CFU/m^2^; mean ± SE)					p-value
	T0	T1	T2	T3	T4	
**Walls**	6.0±1.08 **^a^**	3.3±1.08 **^a^**	0 **^b^**	0 **^b^**	0 **^b^**	0.001
**Floors**	5.4±0.52 **^a^**	5.4 ±0.52 **^a^**	4.5±0.52 **^a^**	1.5±0.52 **^b^**	0 **^b^**	0.001
**Wall-floor junctions**	5.3±0.88 **^a^**	6.8±0.88 **^a^**	2.8±0.88 **^b^**	0 **^c^**	0 **^c^**	0.001

T0: before starting C&D protocol; T1: after the foaming detergent; T2: after carvacrol application; T3: 24 h after the first BP cocktail application; T4: 4 h after the second BP cocktail application. ^a,b,c^: different superscripts indicate significant differences within each sample type over the time points.

Regarding *E. coli*, the results showed a progressive reduction across all three surfaces, with a significant decrease at T1 (after application of the detergent) (p-value < 0.05). The higher reduction was observed at T4 on the wall surface, with an average concentration of 2.7 log_10_ CFU/m². No statistically significant differences were observed between surfaces at T0, T1, and T2 (p-value > 0.05). In contrast, significant differences were detected at T3 and T4 (p-value = 0.002 and 0.001, respectively) (Table 5).

**Table 5:** *E. coli* count at different time points (log_10_ CFU/m^2^), expressed as mean ± standard error (SE).

Surface	Concentration of *E. coli* (log_10_ CFU/m^2^; mean ± SE)					p-value
	T0	T1	T2	T3	T4	
**Walls**	8.8±0.52 **^a^**	7.3 ±0.52 **^ab^**	6.5±0.52 **^b^**	4.0 ±0.52 **^c^**	2.7 ±0.52 **^c^**	0.001
**Floors**	8.9±0.28 **^a^**	7.6±0.28 **^b^**	7.1±0.28 **^bc^**	6.7 ±0.28 **^c^**	6.3±0.28 **^c^**	0.001
**Wall-floor junctions**	8.8±0.38 **^a^**	7.8±0.38 **^ab^**	7.4±0.38 **^b^**	5.9±0.38 **^c^**	5.6±0.38 **^c^**	0.001

T0: before starting C&D protocol; T1: after the foaming detergent; T2: after carvacrol application; T3: 24 h after the first BP cocktail application; T4: 4 h after the second BP cocktail application. ^a,b,c^: different superscripts indicate significant differences within each sample type over the time points.

**Fig. 3:**
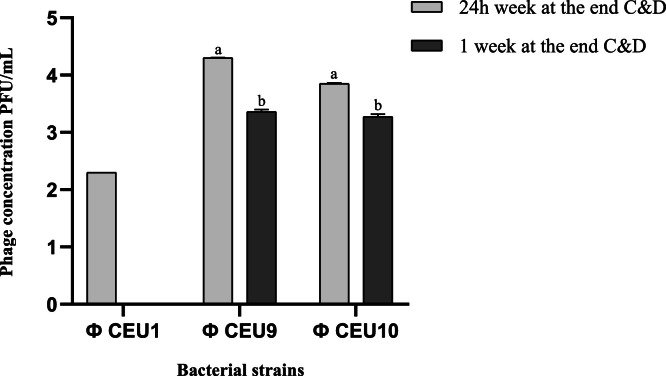
BP count (Ф CEU1: Ф CEU1αP2_0402, Ф CEU9: Ф CEU9αP2_2802, and Ф CEU10: Ф CEU10αP2_2802) in the experimental house 24 h after and one week after the end of the C&D protocol. ^a,b^: different superscripts indicate significant differences within each sample type over the time points.

Regarding the persistence of BP in the environmental samples of the experimental house, results showed a significant reduction in BP count (p-value < 0.05) 24 h after the C&D protocol, and after one week, a complete elimination of phage Ф CEU1αP2_0402. The average phage count after one week was 2.4 log_10_ PFU/mL (Fig. 3).

## Discussion

Nowadays, mST continues to represent one of the major challenges for livestock production systems, particularly in the poultry sector, where it remains one of the leading causes of foodborne infections worldwide (EFSA, 2025). The persistence of *Salmonella* in poultry farms remains a recurring problem, despite the strict implementation of C&D protocols (Samper-Cativiela et al., 2023). This persistence is also linked to the ability of *Salmonella* to form biofilms on farm surfaces, which protect bacterial cells from environmental stress and disinfectants, facilitating long-term survival in poultry facilities (Chylkova et al., 2017; Korzeniowski et al., 2022). Our study demonstrated that the proposed C&D protocol, based on the combination of EO and BP, enabled the complete elimination of mST from the experimentally contaminated environment, highlighting the potential combination of carvacrol and bacteriophages as an alternative approach for surface microbiological control.

The antimicrobial susceptibility profile of the mST strain used in this study revealed resistance to AMP, SME and TET, classifying the strain as MDR. The presence of MDR *Salmonella* strains in poultry production systems has been increasingly reported and represents a major concern for both animal production and public health (Castro-Vargas et al., 2020; Cerqueira-Cézar et al., 2025). In this context, the persistence of these strains in the farm environment assumes particular epidemiological importance, as it may favor the maintenance and spread of the pathogen along the production chain (Cerqueira-Cézar et al., 2025).

In the present study, this epidemiological scenario was reproduced using experimentally infected chickens, which continuously shed mST throughout the entire experimental period without showing clinical signs. This finding further supports the well-documented role of asymptomatic carriers in maintaining environmental contamination in poultry facilities (Velge et al., 2022; Shaji et al., 2023). After the removal of infected animals, in the absence of adequate disinfection, *Salmonella* can persist in the environment at high concentrations (Wang et al., 2023b). In a study conducted by Marin et al. (2011), poultry farms after C&D were shown to have a remaining *Salmonella* contamination rate of 20 % (Marin et al., 2011). In a different study, Papić et al. (2026) reported a *Salmonella* prevalence of 1 % on equipment after in-house disinfection (Papić et al., 2026). In most cases, the main problem is represented by the inadequate use of disinfectants in standard protocols (Marin et al., 2011; Oastler et al., 2022). Indeed, Oastler et al. (2022) demonstrated that in some farms the instructions for the correct use of disinfectants were not followed (Oastler et al., 2022).

However, C&D remain the cornerstone solution to eliminate *Salmonella* from farming environments. Nevertheless, at present, with the increasing restrictions on the use of chemical disinfectants and the growing spread of disinfectant-resistant bacteria, there is a clear demand for complementary, effective, and sustainable control strategies. Among the available alternatives, EOs and BPs are promising options (Di Vito et al., 2020; Wang et al., 2023a; Sevilla-Navarro et al., 2024). Among EOs, carvacrol is one of the most promising compounds against *Salmonella*, in line with previous studies (Nair et al., 2023; Boyer et al., 2024; Cui et al., 2025). Cui et al. (2025) and Boyer et al. (2024) demonstrated a marked antibacterial activity of carvacrol at a concentration of 0.5 mg/mL. Similarly, Nair et al. (2023) showed that carvacrol is able to effectively reduce *Salmonella* contamination at a concentration of 1 %, proving to be more effective than other EOs. These findings were further confirmed by our *in vitro* assays, which showed pronounced antibacterial activity of carvacrol against mST, with complete inhibition of bacterial growth at 0.25 %, comparable to that observed with eugenol and berberine hemisulfate. In the proposed protocol, the choice of the disinfectant focused on carvacrol because, according to the literature, the antibacterial efficacy of eugenol is generally lower or, at best, comparable, but not superior to that of carvacrol, often requiring higher concentrations to achieve the same level of bacterial reduction (Tekin et al., 2025). In addition, carvacrol is a naturally occurring phenolic compound that can be extracted from thyme and, to a greater extent, from oregano, making it more suitable for practical applications (Burt, 2004). Regarding berberine, most available studies focus on its therapeutic mechanisms in poultry rather than on its use as an environmental disinfectant (Yang et al., 2024, 2025). Finally, the three BPs included in the cocktail (Ф CEU1αP2_0402, Ф CEU9αP2_2802, and Ф CEU10αP2_2802) were selected in the present study due to their pronounced lytic activity against mST strains, not only those affecting poultry but also strains associated with livestock production animals such as swine (results pending publication). Moreover, the use of a BP cocktail rather than single isolates was chosen to broaden the spectrum activity against different *Salmonella* serotypes, as well as different mST strains that may be present on farms, thereby enhancing their potential as biocontrol agents. Recent studies have also highlighted the potential of BPs cocktails to control biofilm-associated *Salmonella* contamination; for example, Korzeniowski et al. (2022) demonstrated the complete eradication of *Salmonella* biofilms formed in poultry drinkers using lytic BPs cocktail. Another relevant aspect is the physicochemical stability of the BPs used in this study. The selected phages showed stability across a wide pH range, including both acidic and alkaline conditions. This characteristic allowed their integration into the C&D protocol in association with the alkaline detergent (Hyprelva Net Plus, 3 %) used during the sanitation phase. Such compatibility with detergents or disinfectants is particularly important for practical applications in farm environments, as it allows their combination with various chemical biocides commonly used in poultry production (Stachler et al., 2021). In addition, the BP showed good stability at low temperatures, allowing their storage under refrigeration without a significant loss of infectivity. This feature facilitates their handling and preservation prior to application in farm environments. Conversely, phage titers showed a progressive reduction at temperatures approaching 60 °C, while complete inactivation was observed at temperatures above 80 °C, indicating that their application should preferably occur after the use of hot water during cleaning procedures. However, considering that some cleaning protocols involve water temperatures around 60 °C, the observed thermal tolerance suggests that phages could still be compatible with such procedures (Gentile et al., 2025). Finally, the one-step growth analysis provided insight into the replication dynamics of the selected bacteriophages. The relatively short eclipse period, approximately 20–30 minutes, indicates a rapid infection cycle in which phages penetrate the bacterial cell, replicate, and lyse the host within a short time frame (Abedon et al., 2001). This rapid lytic cycle represents a particularly relevant advantage for environmental biocontrol strategies, as it allows a fast reduction of the target bacterial population following application.

Following selection of the compounds, an innovative C&D protocol was subsequently developed. Based on the available evidence in the literature, this study represents the first protocol to evaluate the combination between an EO and a BP cocktail under conditions simulating a real farm environment. In particular, the proposed protocol consists of five sequential phases, ranging from the removal of organic matter to the application of disinfectants, in accordance with standard C&D protocols widely described in the literature (Luyckx et al., 2015; Martelli et al., 2017; De Lorenzi et al., 2020; Papić et al., 2026).

The first step, consisting of mechanical cleaning followed by detergent application, proved to be essential for reducing organic matter on surfaces. However, it has been demonstrated in this study that when detergent is applied alone, this step was essential but insufficient to ensure complete elimination of *Salmonella* from contaminated surfaces, with an average *Salmonella* prevalence on the three surfaces of 4.4 log_10_ CFU/m². Hansson et al. (2025) reported that, among ten farms tested, the only one that did not use a detergent showed a mean bacterial load of 600 CFU, compared with the others (< 100 CFU) (Hassanzadeh et al., 2024). Similarly, Walia et al. (2017) reported that the use of detergent alone resulted in a *Salmonella* prevalence of 54 % on the analyzed surfaces, which was significantly higher than that observed when the detergent was used in combination with a disinfectant (Walia et al., 2017). In addition, the assessment of the general hygienic status of the environment, based on the mean *E. coli* counts, revealed overall poor hygienic conditions after only the first C&D step, with a mean of 7.5 log₁₀ CFU/m². This result indicates that a single intervention is not sufficient to ensure an adequate level of environmental hygiene, highlighting the need to integrate additional steps into the C&D protocol (Walia et al., 2017; Sevilla-Navarro et al., 2024; Hansson et al., 2025).

The second step consists of the application of the disinfectant, in this case, replaced by the EO carvacrol. To the best of our knowledge, this is the first study to report the use of carvacrol as a disinfectant in an experimental poultry farming setting. In agreement with the *in vitro* results, the application of carvacrol as a disinfectant led to a significant reduction in mST detection on walls and wall-floor junctions (6.5 log₁₀ CFU/m² and 1.53 log₁₀ CFU/m², respectively), except for the floor (0.74 log₁₀ CFU/m²), which represents the surface most exposed to the accumulation of organic residues, thereby favoring microbial persistence (Walia et al., 2017). In addition, a reduction in environmental *E. coli* levels was also observed (<0.57 log_10_ CFU/m²), suggesting a beneficial effect of the treatment on the general hygienic status of the environment.

In order to achieve the complete elimination of mST from surfaces, the final step of the protocol involved the application of a BP cocktail at a 24 h interval. In particular, the double application of the phage cocktail allowed the complete elimination of *Salmonella* from all sampled surfaces already after the first phage treatment, with a particularly pronounced effect on walls and wall-floor junctions (0.50 log₁₀ CFU/m² and 0 log₁₀ CFU/m²), compared to the application of carvacrol alone. Similar results were reported by Sevilla-Navarro et al. (2024), who observed complete elimination of *Salmonella* after the second application of a phage cocktail. (Sevilla-Navarro et al., 2024). This outcome can be attributed to the intrinsic characteristics of BP, including their high specificity for the target strain and their ability to replicate in the presence of the bacterial host, locally amplifying their antimicrobial effect even in microenvironments that are difficult to reach (Sillankorva et al., 2012). Moreover, the use of a phage cocktail rather than single isolates allows for a broader activity spectrum.

Another relevant aspect concerns the environmental persistence of BP. In the present study, phages showed a slight reduction one week after the application of the C&D protocol. This characteristic represents an advantage in terms of biosecurity, as it ensures an effective long-term environmental antimicrobial activity, particularly during the early stages of the production cycle, when animals are more susceptible to infections (Jordá et al., 2023). Similarly, Kuźmińska-Bajor et al. (2025) demonstrated that the BP cocktail administered to the litter to control multidrug-resistant *E. coli* maintained good stability and persistence throughout the entire experimental period, confirming the ability of phages to remain active in complex environmental settings (Kuźmińska-Bajor et al., 2025). In addition, the safety of BP for animals, workers, and the environment is well documented, and several phage-based products are already approved for use in the food and agricultural sectors, further reinforcing the practical feasibility of this approach (Wójcicki et al., 2025). For instance, in the United States, phage-based products have been commercially available for several years for the control of bacterial plant diseases (AgriPhage) (Wójcicki et al., 2025). In contrast, in Europe, BP commercialization is still limited, mainly due to a more restrictive regulatory framework. Nevertheless, authorized products such as BioLyse-PB and Erwiphage Plus are already available on the market for the control of bacterial plant diseases (Wójcicki et al., 2025). In parallel, research is increasingly focusing on the development of BP applications in the livestock sector, particularly as feed additives. Recently, a bacteriophage-based feed additive for *Salmonella* control in the poultry sector (BAFASAL) has been approved in Poland (Wójcicki et al., 2025). However, in Europe, there are no phage-based products authorized for the disinfection of farm surfaces.

## Conclusions

This study demonstrates that the potential combination of carvacrol and a BP cocktail, applied within a C&D protocol, represents an effective and sustainable strategy for controlling mST in poultry environments. The integrated approach enabled not only the complete elimination of the target pathogen but also an overall improvement in surface hygienic conditions. Moreover, the persistence of BP in the environment even after disinfection supports their potential role as a biocontrol tool, contributing to the long-term protection of poultry farming environments against *Salmonella*. Overall, the results indicate that the proposed protocol may represent a valid alternative to conventional chemical disinfectants, in line with current demands for sustainability, safety, and reduced use of biocides in the livestock sector.

## Availability of data and materials

All data generated or analyzed during this study are included in this published article.

## Funding

This study was funded by University CEU-UCH (INDI 25/48 and GIR 25/37).

## CRediT authorship contribution statement

**N. Gentile:** Writing – review & editing, Writing – original draft, Visualization, Data curation, Conceptualization. **L. Montoro-Dasi:** Writing – review & editing, Visualization. **A. Marco-Fuertes:** Writing – review & editing, Visualization. **P.J. Marín-García:** Writing – original draft. **P. Domingo-Calap:** Writing – review & editing, Visualization. **L. Lorenzo-Rebenaque:** Writing – review & editing, Writing – original draft, Visualization, Conceptualization. **C. Marin:** Writing – review & editing, Visualization, Conceptualization.

## Disclosures

The authors declare that they have no known competing financial interests or personal relationships that could have appeared to influence the work reported in this paper.

## Appendix Supplementary materials

Image, application 1

## Footnotes

Supplementary material associated with this article can be found, in the online version, at doi:10.1016/j.psj.2026.107124.

## References

[bib0001] Abedon S.T., Herschler T.D., Stopar D. (2001). Bacteriophage latent-period evolution as a response to resource availability. Appl. Environ. Microbiol..

[bib0002] Bankevich A., Nurk S., Antipov D., Gurevich A.A., Dvorkin M., Kulikov A.S., Lesin V.M., Nikolenko S.I., Pham S., Prjibelski A.D., Pyshkin A.V., Sirotkin A.V., Vyahhi N., Tesler G., Alekseyev M.A., Pevzner P.A. (2012). SPAdes: a new genome assembly algorithm and its applications to single-cell sequencing. J. Comput. Biol..

[bib0003] Bezek K., Avberšek J., Rojs O.Z., Barlič-Maganja D. (2023). Antimicrobial and antibiofilm effect of commonly used disinfectants on Salmonella infantis isolates. Microorganisms.

[bib0004] Bouras G., Nepal R., Houtak G., Psaltis A.J., Wormald P.-J., Vreugde S. (2023). Pharokka: a fast scalable bacteriophage annotation tool (T Marschall, Ed.). Bioinformatics.

[bib0005] Boyer E., Galán-Relaño Á., Romero-Salmoral A., Barraza P., Gómez-Gascón L., Tarradas C., Luque I., De Aguiar F.C., Lorenzo B.H. (2024). Post-Antibiotic and post-antibiotic sub-minimum inhibitory concentration effects of carvacrol against Salmonella typhimurium. Animals.

[bib0006] Burt S. (2004). Essential oils: their antibacterial properties and potential applications in foods—a review. Int. J. Food Microbiol..

[bib0007] Campana R., Baffone W. (2018). Carvacrol efficacy in reducing microbial biofilms on stainless steel and in limiting re-growth of injured cells. Food Control.

[bib0008] Castro-Vargas, R., H-S MP, R-H R, and R-B IS. 2020. Antibiotic resistance in Salmonella spp. isolated from poultry: a global overview. 13:2070–2084. 10.14202/vetworld.2020.2070-2084 PMC7704309 33281339

[bib0009] Cerqueira-Cézar C.K., Sampaio A.N.D.C.E., Caron E.F.F., Dellaqua T.T., Ribeiro L.F.M., Tadielo L.E., Pantoja J.C.D.F., Viana G.G.F., Rossi G.A.M., Spanu C., Possebon F.S., Pereira J.G. (2025). Antimicrobial resistance in chicken meat: comparing Salmonella, Escherichia coli, and Enterococcus from conventional and antibiotic-free productions. Microorganisms.

[bib0010] Chen S., Zhou Y., Chen Y., Gu J. (2018). fastp: an ultra-fast all-in-one FASTQ preprocessor. Bioinformatics.

[bib0011] Chouhan S., Sharma K., Guleria S. (2017). Antimicrobial activity of some essential oils—present status and future perspectives. Medicines.

[bib0012] Chylkova T., Cadena M., Ferreiro A., Pitesky M. (2017). Susceptibility of Salmonella biofilm and planktonic bacteria to common disinfectant agents used in poultry processing. J. Food Prot..

[bib0013] Clausen, P. T. L. C., F. M. Aarestrup, and O. Lund. 2018. Rapid and precise alignment of raw reads against redundant databases with KMA. 19 Available at https://link.springer.com/article/10.1186/s12859-018-2336-6. 10.1186/s12859-018-2336-6 PMC6116485 30157759

[bib0014] Cui H., Chen X., Aziz T., Mohamed R.A.E.H., Al-Asmari F., Alshammari J.M., Al-Joufi F.A., Shi C., Lin L. (2025). Inactivation mechanisms of carvacrol on Salmonella typhimurium and its combined inhibitory effects with blue light-405 nm for chicken meat preservation. Int. J. Food Microbiol..

[bib0015] De Lorenzi G., Borella L., Alborali G.L., Prodanov-Radulović J., Štukelj M., Bellini S. (2020). African swine fever: a review of cleaning and disinfection procedures in commercial pig holdings. Res. Vet. Sci..

[bib0016] Di Vito M., Cacaci M., Barbanti L., Martini C., Sanguinetti M., Benvenuti S., Tosi G., Fiorentini L., Scozzoli M., Bugli F., Mattarelli P. (2020). Origanum vulgare Essential oil vs. a commercial mixture of essential oils: in vitro effectiveness on Salmonella spp. from poultry and swine intensive livestock. Antibiotics.

[bib0017] Domingo-Calap P., Beamud B., Vienne J., González-Candelas F., Sanjuán R. (2020). Isolation of four lytic phages infecting Klebsiella pneumoniae K22 clinical isolates from Spain. Int. J. Mol. Sci..

[bib0018] EFSA. 2025. The European Union One Health 2024 zoonoses report. 10.2903/j.efsa.2025.9759 PMC12686834 41377167

[bib0019] EUCAST. 2024. The European Committee on antimicrobial susceptibility testing. Breakpoint tables for interpretation of MICs and zone diameters. Available at https://www.eucast.org/fileadmin/src/media/PDFs/EUCAST_files/Breakpoint_tables/v_14.0_Breakpoint_Tables.pdf.

[bib0020] European Commission. 2003. Regulation (EC) No 2160/2003 of the European Parliament and of the Council of 17 November 2003 on the control of salmonella and other specified food-borne zoonotic agents.

[bib0021] European Commission. 2009. Regulation (EC) No 1223/2009 of the European Parliament and of the Council of 30 November 2009 on cosmetic products.

[bib0022] European Pharmacopoeia (EP): 04/ 2013:2520.

[bib0023] European Pharmacopoeia (EP): 04/ 2013:2521.

[bib0024] Gentile N., Lorenzo-Rebenaque L., Marco-Fuertes A., Montoro-Dasi L., Marin C. (2025). Emerging challenges in Salmonella control: the need for innovative and sustainable disinfection strategies in poultry farming. Pathogens.

[bib0025] Gilchrist C.L.M., Chooi Y.-H. (2021). clinker & clustermap.js: automatic generation of gene cluster comparison figures (P Robinson, Ed.). Bioinformatics.

[bib0026] GitHub. 2025. GitHub - lh3/seqtk: toolkit for processing sequences in FASTA/Q formats. Available at https://github.com/lh3/seqtk.

[bib0027] Gvaladze T., Lehnherr H., Hertwig S. (2024). A bacteriophage cocktail can efficiently reduce five important Salmonella serotypes both on chicken skin and stainless steel. Front. Microbiol..

[bib0028] Hansson I., Dzieciolowski T., Rydén J., Boqvist S. (2025). Evaluation of cleaning and disinfection procedures on poultry farms. Poult. Sci..

[bib0029] Hassanzadeh M., Mirzaie S., Pirmahalle F.R., Yahyaraeyat R., Razmyar J. (2024). Effects of thyme (Thymus vulgaris) essential oil on bacterial growth and expression of some virulence genes in Salmonella enterica serovar enteritidis. Vet. Med. Sci..

[bib0030] Jordá J., Lorenzo-Rebenaque L., Montoro-Dasi L., Marco-Fuertes A., Vega S., Marin C. (2023). Phage-based biosanitation strategies for minimizing persistent Salmonella and campylobacter bacteria in poultry. Animals.

[bib0031] Korzeniowski P., Śliwka P., Kuczkowski M., Mišić D., Milcarz A., Kuźmińska-Bajor M. (2022). Bacteriophage cocktail can effectively control Salmonella biofilm in poultry housing. Front. Microbiol..

[bib0032] Kumbhar, V. R., and S. B. Geddugol. 2025. Prevalence of health effects due to disinfectant exposure and its impact on selected physiological parameters among Class D workers: a descriptive cross-sectional study. Cureus Available at https://www.cureus.com/articles/330304-prevalence-of-health-effects-due-to-disinfectant-exposure-and-its-impact-on-selected-physiological-parameters-among-class-d-workers-a-descriptive-cross-sectional-study (verified 30 October 2025). 10.7759/cureus.79994 PMC11965772 40182389

[bib0033] Kuźmińska-Bajor M., Kuczkowski M., Konkol D., Korczyński M., Rakicka-Pustułka M., Kozioł S., Tomaszewska-Hetman L., Rywińska A. (2025). Environmental application of a bacteriophage cocktail reduces antibiotic-resistant Escherichia coli in poultry litter without disrupting gut microbiota. Animals.

[bib0034] Li, H., and R. Durbin. 2009. Fast and accurate short read alignment with Burrows–Wheeler transform. 25:1754–1760. 10.1093/bioinformatics/btp324 PMC2705234 19451168

[bib0035] Li H., Handsaker B., Wysoker A., Fennell T., Ruan J., Homer N., Marth G., Abecasis G., Durbin R., 1000 Genome Project Data Processing Subgroup (2009). The sequence alignment/map format and SAMtools. Bioinformatics.

[bib0036] Luyckx K.Y., Van Weyenberg S., Dewulf J., Herman L., Zoons J., Vervaet E., Heyndrickx M., De Reu K. (2015). On-farm comparisons of different cleaning protocols in broiler houses. Poult. Sci..

[bib0037] Maillard J.-Y., Pascoe M. (2024). Disinfectants and antiseptics: mechanisms of action and resistance. Nat. Rev. Microbiol..

[bib0038] Marcelino, V. R., P. T. L. C. Clausen, J. P. Buchmann, M. Wille, J. R. Iredell, W. Meyer, O. Lund, T. C. Sorrell, and E. C. Holmes. 2020. CCMetagen: comprehensive and accurate identification of eukaryotes and prokaryotes in metagenomic data. 21 Available at https://link.springer.com/article/10.1186/s13059-020-02014-2. 10.1186/s13059-020-02014-2 PMC7189439 32345331

[bib0039] Marco-Fuertes A., Jordá J., Marin C., Lorenzo-Rebenaque L., Montoro-Dasi L., Vega S. (2023). Multidrug-resistant Escherichia coli strains to last resort human antibiotics isolated from healthy companion animals in Valencia region. Antibiotics.

[bib0040] Marin C., Balasch S., Vega S., Lainez M. (2011). Sources of Salmonella contamination during broiler production in Eastern Spain. Prev. Vet. Med..

[bib0042] Marin C., Torres C., Marco-Jiménez F., Cerdà-Cuéllar M., Sevilla S., Ayats T., Vega S. (2018). Supplementary feeding stations for conservation of vultures could be an important source of monophasic Salmonella typhimurium 1,4,[5],12:i:-. Sci. Total Environ..

[bib0043] Martelli F., Lambert M., Butt P., Cheney T., Tatone F.A., Callaby R., Rabie A., Gosling R.J., Fordon S., Crocker G., Davies R.H., Smith R.P. (2017). Evaluation of an enhanced cleaning and disinfection protocol in Salmonella contaminated pig holdings in the United Kingdom (P Butaye, Ed.). PLoS One.

[bib0044] Moreno Switt A., Sulakvelidz A., Wiedmann M., Kropinski A.M., Wishart D.S., Poppe C., Yongjie L. (2015).

[bib0045] Nair D.V.T., Manjankattil S., Peichel C., Martin W., Donoghue A.M., Venkitanarayanan K., Kollanoor Johny A. (2023). Effect of plant-derived antimicrobials, eugenol, carvacrol, and β-resorcylic acid against Salmonella on organic chicken wings and carcasses. Poult. Sci..

[bib0046] Nayfach S., Camargo A.P., Schulz F., Eloe-Fadrosh E., Roux S., Kyrpides N.C. (2021). CheckV assesses the quality and completeness of metagenome-assembled viral genomes. Nat. Biotechnol..

[bib0047] Oastler C.E., Nichols C., Newton K., Cawthraw S., Gosling R.J., Martelli F., Wales A.D., Davies R.H. (2022). Observations on the distribution and control of *Salmonella* in commercial broiler hatcheries in Great Britain. Zoonoses Public Health.

[bib0048] Obe T., Kiess A.S., Nannapaneni R. (2024). Antimicrobial tolerance in salmonella: contributions to survival and persistence in processing environments. Animals.

[bib0049] Papić B., Mićunović J., Slavec B., Šemrov N., Rojs O.Z., Avberšek J. (2026). A case study of critical points for the entry and spread of Salmonella Infantis in a broiler farm. Ir. Vet. J..

[bib0050] Papoula-Pereira R., Alvseike O., Cenci-Goga B.T., Grispoldi L., Nagel-Alne G.E., Ros-Lis J.V., Thomas L.F. (2025). Economic evidence for the control of Salmonella in animal-derived food systems: a scoping review. Food Control.

[bib0051] Parsons B.N., Crayford G., Humphrey T.J., Wigley P. (2013). Infection of chickens with antimicrobial-resistant *Salmonella enterica* Typhimurium DT193 and monophasic *Salmonella* Typhimurium-like variants: an emerging risk to the poultry industry?. Avian Pathol..

[bib0052] Quinlan, A. R. 2014. BEDTools: the Swiss-army tool for genome feature analysis. 47. 10.1002/0471250953.bi1112s47 PMC4213956 25199790

[bib0053] Samper-Cativiela C., Prieto M.E., Collado S., De Frutos C., Branscum A.J., Saez J.L., Alvarez J. (2023). Risk factors for Salmonella detection in commercial layer flocks in Spain. Animals.

[bib0054] Sevilla-Navarro S., Torres-Boncompte J., Garcia-Llorens J., Bernabéu-Gimeno M., Domingo-Calap P., Catalá-Gregori P. (2024). Fighting Salmonella Infantis: bacteriophage-driven cleaning and disinfection strategies for broiler farms. Front. Microbiol..

[bib0055] Shaji S., Selvaraj R.K., Shanmugasundaram R. (2023). Salmonella infection in poultry: a review on the pathogen and control strategies. Microorganisms.

[bib0056] Sillankorva S.M., Oliveira H., Azeredo J. (2012). Bacteriophages and their role in food safety. Int. J. Microbiol..

[bib0057] Stachler E., Kull A., Julian T.R. (2021). Bacteriophage treatment before chemical disinfection can enhance removal of plastic-surface-associated Pseudomonas aeruginosa (AJ McBain, Ed.). Appl. Environ. Microbiol..

[bib0058] Tekin Y., Yazgan H., Gokmen T.G., Gungor N., Uprak N.S. (2025). Integrated analysis of Salmonella infantis in chicken meat: epidemiological surveillance, antibiotic resistance, and potential bioactive control agents. Pathogens.

[bib0059] Tong C., Hu H., Chen G., Li Z., Li A., Zhang J. (2021). Disinfectant resistance in bacteria: mechanisms, spread, and resolution strategies. Environ. Res..

[bib0060] Velge P., Menanteau P., Chaumeil T., Barilleau E., Trotereau J., Virlogeux-Payant I., Virulence Bacterial, Gal-Mor O. (2022). Methods in Molecular Biology.

[bib0061] Walia K., Argüello H., Lynch H., Grant J., Leonard F.C., Lawlor P.G., Gardiner G.E., Duffy G. (2017). The efficacy of different cleaning and disinfection procedures to reduce Salmonella and Enterobacteriaceae in the lairage environment of a pig abattoir. Int. J. Food Microbiol..

[bib0062] Wang W., Li T., Chen J., Ye Y. (2023). Inhibition of Salmonella enteritidis by essential oil components and the effect of storage on the quality of chicken. Foods.

[bib0063] Wang J., Vaddu S., Bhumanapalli S., Mishra A., Applegate T., Singh M., Thippareddi H. (2023). A systematic review and meta-analysis of the sources of Salmonella in poultry production (pre-harvest) and their relative contributions to the microbial risk of poultry meat. Poultry Sci..

[bib0064] Wang Y., Xu X., Jia S., Qu M., Pei Y., Qiu S., Zhang J., Liu Y., Ma S., Lyu N., Hu Y., Li J., Zhang E., Wan B., Zhu B., Gao G.F. (2025). A global atlas and drivers of antimicrobial resistance in Salmonella during 1900-2023. Nat. Commun..

[bib0065] Wińska K., Mączka W., Łyczko J., Grabarczyk M., Czubaszek A., Szumny A. (2019). Essential oils as antimicrobial agents—myth or real alternative?. Molecules.

[bib0066] Wójcicki M., Sokołowska B., Górski A., Jończyk-Matysiak E. (2025). Dual nature of bacteriophages: friends or foes in minimally processed food products—a comprehensive review. Viruses.

[bib0067] Yang L., Qiao Y., Chen R., Wang M., Zhou B., Zhang X., Zhao Y., Shi H., Xia L., Fu Q. (2025). Evaluation of the therapeutic effect of Berberine microcapsules on Salmonella enteritidis-infected mice. BMC Vet. Res..

[bib0068] Yang L., Sun J., Yang T., Zhang X., Xu C., Wei Y., Li Y., Zhao Y., Zhang S., Wu Q., Shi H., Fu Q., Xia L. (2024). Therapeutic effects and mechanisms of berberine on enteritis caused by Salmonella in poultry. Front. Microbiol..

