# Supplementary material for: Combining bacteriophages and essential oils for the elimination of monophasic Salmonella Typhimurium and evaluation of phage persistence in poultry farm environments
Source: Poult Sci. 2026 May 13;105(9):107124. doi: 10.1016/j.psj.2026.107124 (PMC13226889; doi:10.1016/j.psj.2026.107124)
Supplement: Supplementary file 1 [file mmc1.docx]

| **Antimicrobial** | **Abbreviation** | **Susceptible**  **(≤ mg/L)** | **Resistant**  **(> mg/L)** |
| --- | --- | --- | --- |
| *Amikacin* | AMI | 8 | 8 |
| *Ampicillin* | AMP | 8 | 8 |
| *Azithromycin* | AZI | 16 | 16 |
| *Cefotaxime* | CTA | 1 | 2 |
| *Ceftazidime* | CTZ | 1 | 4 |
| *Chloramphenicol* | CHL | 8 | 16 |
| *Ciprofloxacin* | CIP | 0.06 | 0.06 |
| *Colistin* | COL | 2 | 2 |
| *Gentamicin* | GEN | 2 | 2 |
| *Meropenem* | MER | 2 | 8 |
| *Nalidixic acid* | NAL | 8 | 8 |
| *Sulfamethoxazole* | SME | 64 | 64 |
| *Tetracycline* | TET | 8 | 8 |
| *Tigecycline* | TIG | 0.5 | 0.5 |
| *Trimethoprim* | TRI | 4 | 4 |

**Table S1.** The antimicrobial panel and corresponding EUCAST *v.*14.0 breakpoint concentrations (EUCAST, 2024).
